# PREFUL MRI Depicts Dual Bronchodilator Changes in COPD: A Retrospective Analysis of a Randomized Controlled Trial

**DOI:** 10.1148/ryct.210147

**Published:** 2022-04-21

**Authors:** Andreas Voskrebenzev, Till F. Kaireit, Filip Klimeš, Gesa H. Pöhler, Lea Behrendt, Heike Biller, Korbinian Berschneider, Frank Wacker, Tobias Welte, Jens M. Hohlfeld, Jens Vogel-Claussen

**Affiliations:** From the Institute for Diagnostic and Interventional Radiology (A.V., T.F.K., F.K., G.H.P., L.B., F.W., J.V.C.) and Department of Respiratory Medicine (T.W., J.M.H.), Hannover Medical School, Carl-Neuberg-Str 1, 30625 Hannover, Germany; German Center for Lung Research (BREATH), Hannover, Germany (A.V., T.F.K., F.K., G.H.P., L.B., H.B., F.W., T.W., J.M.H., J.V.C.); Fraunhofer Institute for Toxicology and Experimental Medicine, Hannover, Germany (H.B., J.M.H.); and Novartis Pharma, Clinical Research Respiratory, Nuremberg, Germany (K.B.).

**Keywords:** MRI, COPD, Perfusion, Ventilation, Lung, Pulmonary

## Abstract

**Purpose:**

To assess whether dynamic ventilation and perfusion (Q) biomarkers derived by phase-resolved functional lung (PREFUL) MRI can measure treatment response to 14-day therapy with indacaterol-glycopyrronium (IND-GLY) and correlate to clinical outcomes including lung function, symptoms, and cardiac function in patients with chronic obstructive pulmonary disease (COPD), as determined by spirometry, body plethysmography, cardiac MRI, and dyspnea score measurements.

**Materials and Methods:**

The cardiac left ventricular function in COPD (CLAIM) study enrolled patients aged 40 years or older with COPD, stable cardiovascular function, and hyperinflation (residual volume > 135% predicted). Dynamic MRI data of these patients were retrospectively analyzed using the PREFUL technique to assess the effect of 14-day IND-GLY treatment versus placebo on regional measurements of ventilation dynamics. After manual segmentation of the lung parenchyma, flow-volume loops of each voxel were correlated to an individualized reference flow-volume loop, creating a two-dimensional flow-volume loop correlation map (FVL-CM) as a measure of ventilation dynamics. Ventilation-perfusion match (VQM) was evaluated in combination with perfusion and regional ventilation (VQM_RVent_) and with perfusion and the FVL-CM measurement (VQM_CM_). For image and statistical analysis, the lung parenchyma was segmented as a region of interest by manually delineating the lung boundary and excluding the large (central) vessels for each section. Differences in ventilation, perfusion, and VQM between IND-GLY and placebo were compared using analysis of variance, with study treatment, patient, and period included as factors.

**Results:**

Fifty patients (mean age, 64.3 years ± 7.65 [SD]; 35 men) were included in this analysis. IND-GLY significantly increased mean correlation as measured with FVL-CM versus that of placebo (least squares [LS] means treatment difference: 0.05 [95% CI: 0.03, 0.07]; *P* < .0001). Compared with placebo, IND-GLY increased mean Q (LS means treatment difference: 9.27 mL/min/100 mL [95% CI: 0.05, 18.49]; *P* = .049) and improved both VQM_CM_ and VQM_RVent_ (LS means treatment difference: 0.06 [95% CI: 0.03, 0.08]; *P* < .0001 and 0.05 [95% CI: 0.02, 0.08]; *P* = .001, respectively).

**Conclusion:**

Regional ventilation dynamics and VQM measured by PREFUL MRI show treatment response in COPD.

*Supplemental material is available for this article*.

Clinical trial registration no. NTR6831

**Keywords:** MRI, COPD, Perfusion, Ventilation, Lung, Pulmonary

Published under a CC BY 4.0 license

## Graphical abstract

**Figure:**
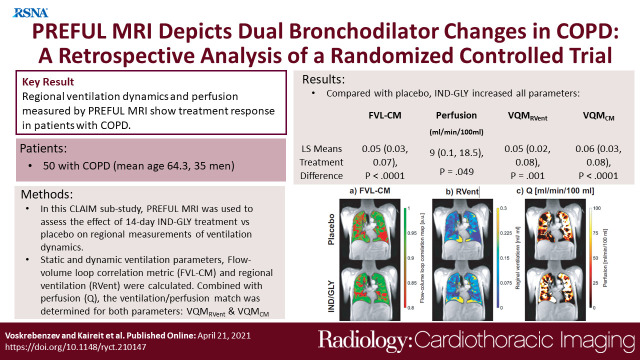

**Summary**

Indacaterol-glycopyrronium improves ventilation, perfusion, and ventilation-perfusion match in patients with chronic obstructive pulmonary disease measured by phase-resolved functional lung (PREFUL) MRI; therefore, PREFUL could provide end points in future cardiopulmonary trials.

**Key Points**

■ Compared with placebo, indacaterol-glycopyrronium (IND-GLY) increased mean flow-volume loop correlation (least squares [LS] mean treatment difference: 0.05 [95% CI: 0.03, 0.07]; *P* < .0001) and reduced ventilation heterogeneity (LS means treatment difference: –0.05 [95% CI: –0.07, –0.03]; *P* < .0001).
■ Compared with placebo, IND-GLY increased mean perfusion (Q) (LS means treatment difference: 9 mL/min/100 mL [95% CI: 0.1, 18.5]; *P* = .049).
■ Compared with placebo, IND-GLY increased ventilation-perfusion match (VQM) with Q and regional ventilation (VQM_RVent_) (LS means treatment difference: 0.05 [95% CI: 0.02, 0.08]; *P* = .001) and VQM with Q and flow-volume loop correlation map (VQM_CM_) (LS means treatment difference: 0.06 [95% CI: 0.03, 0.08]; *P* < .0001).

## Introduction

Chronic airflow obstruction is a feature of chronic obstructive pulmonary disease (COPD) causing substantial morbidity and mortality worldwide in affected patients (1). Therefore, sensitive treatment monitoring is desirable for optimized individual patient management. Several functional lung MRI techniques have been developed to monitor chronic lung disease, of which Fourier decomposition and related techniques show promise. Fourier decomposition MRI is a dynamic, free-breathing, non–contrast-enhanced technique based on the registration of a time series of images, followed by voxelwise frequency analysis (2). It provides a means to measure pulmonary ventilation, perfusion (Q), and ventilation-perfusion match (VQM) without the use of potentially harmful tracer gases, contrast media, or ionizing radiation (2). Ventilation values derived from this technique correlate with those derived from hyperpolarized gas MRI (3) and dynamic fluorinated gas washout MRI (4) in patients with COPD, offering a potential alternative imaging technique.

Calculation of the final parameters in Fourier decomposition effectively involves only two respiratory and cardiac phases, leading to substantial information loss. To address this problem, the postprocessing algorithm phase-resolved functional lung (PREFUL) MRI was recently developed to quantify perfusion and ventilation dynamics (5,6). The concept of ventilation- and perfusion-phase sorting as an addition to Fourier decomposition analysis was previously introduced as SELf-gated Non–Contrast-Enhanced Functional Lung imaging (SENCEFUL) (7). Although this approach can sort each phase-encoding step, a special sequence with non–phase-encoded direct current signal acquisition is mandatory. On the contrary, the phase sorting used in PREFUL can be used in conjunction with default sequences (spoiled gradient-echo or balanced steady-state free precession). Preliminary results in patients with COPD confirm improved correlation of spirometric lung function (forced expiratory volume in 1 second [FEV_1_] percent predicted) with the dynamic regional flow-volume parameters compared to the static regional ventilation (RVent) parameter (8). Similarly, analysis of dynamic regional flow-volume loop parameters was shown to be sufficiently sensitive for the detection of early chronic lung allograft dysfunction (9).

PREFUL MRI–derived perfusion defect percentages have been shown to correlate with dynamic contrast-enhanced–derived pulmonary microvascular blood flow perfusion defect percentages in a prospective study in patients with COPD (10). Both ventilation and perfusion PREFUL MRI parameters were repeatable over two scan sessions in both healthy controls and patients with COPD (11).

In the cardiac left ventricular function in COPD (CLAIM) study, the RVent parameter showed a significant treatment response to 14-day therapy with the dual bronchodilator indacaterol-glycopyrronium (IND-GLY) (12). Posttreatment metrics of pulmonary microvascular blood flow and RVent correlated with posttreatment left ventricular end-diastolic volume and Transition Dyspnea Index (TDI) values but were not correlated with treatment change (12). It is unknown whether IND-GLY also improves regional VQM and RVent dynamics. The PREFUL MRI parameters in this work were not available at the time of the original CLAIM study, and this retrospective analysis should evaluate if these promising PREFUL ventilation-perfusion parameters are suited as reliable markers in future trials.

Therefore, we hypothesize that the regional information of PREFUL MRI–derived flow-volume loops, PREFUL perfusion, and the combined ventilation-perfusion metrics make this a sensitive method for monitoring patients with COPD. If proven, PREFUL MRI may have added value compared with lung function testing, dyspnea scores, and anatomic chest CT. The purpose of this retrospective CLAIM substudy was to assess whether dynamic ventilation and perfusion biomarkers derived by PREFUL MRI can measure treatment response to 14-day therapy with IND-GLY and correlate to clinical outcomes including lung function, symptoms, and cardiac function in patients with COPD, as determined by spirometry, body plethysmography, cardiac MRI, and dyspnea score measurements.

## Materials and Methods

### Participants

This study is a retrospective analysis of data prospectively acquired during the CLAIM study (ClinicalTrials.gov identifier: NCT02442206), which took place between May 18, 2015, and April 20, 2017. All patients included in this study were reported previously: the primary, secondary, and exploratory end points of the CLAIM study are published (12,13). CLAIM study participants were patients aged 40 years or older with a clinical diagnosis of COPD, stable cardiovascular function, and baseline hyperinflation (residual volume > 135% predicted), smoking history ≥ 10 pack-years, and airflow limitation (baseline postbronchodilator FEV_1_ of less than 80% predicted and a postbronchodilator FEV_1_-to–forced vital capacity ratio of less than 0.7). Patients with arrhythmias, heart failure (left ventricular ejection fraction < 40%), unstable ischemic heart disease, or uncontrolled hypertension were excluded. Patients who discontinued the study were not replaced. Further details of inclusion and exclusion criteria have been previously described (13). The population was selected by the investigators after completion of the CLAIM study, who stayed blinded to placebo and treatment periods even after completion of the study. Patients were included in this analysis if they had completed the whole MRI examination at all four visits of the CLAIM study and had no protocol deviations or missing data that precluded a precise analysis using this method. Patient race was recorded to measure potential lack of diversity in a single-center trial in a small population. Race was investigator observed. CLAIM was approved by the ethics committee of Hannover Medical School and the German Federal Institute for Drugs and Medical Devices. All patients provided written informed consent.

### Study Design

The CLAIM study was a randomized, double-blind, placebo-controlled, crossover study (13) that assessed the effect of 14-day treatment with once-daily IND-GLY in patients with COPD with hyperinflated lungs. Patients received once-daily IND-GLY (110/50 µg) for 14 days followed by placebo for 14 days, or vice versa, with the two treatment periods separated by a 14-day washout.

### MRI Procedure

Participants underwent PREFUL MRI with a 1.5-T scanner (Magnetom Avanto; Siemens Healthcare) under free breathing for 1 minute per section in a head-first supine position. Three strictly coronal sections (one at the middle of the tracheal level, one anterior to the trachea, and one posterior to the trachea) with a 11.25-mm section gap were acquired. To achieve a high reproducibility of the middle section, a transversal localizer was used to find the tracheal bifurcation. Image acquisition was performed with the following acquisition parameters: spoiled gradient-echo sequence at a temporal resolution of 288 msec with echo time of 0.82 msec, repetition time of 3 msec, flip angle of 5°, matrix size of 128 × 96 interpolated with zero filling to 256 × 256, field of view of 50 × 50 cm^2^, section thickness of 15 mm, gap between sections of 11.25 mm, and pixel bandwidth of 1500 Hz/pixel. See Appendix E1 (supplement) for cardiac MRI and dynamic contrast-enhanced MRI methods and Table E4 (supplement) for MRI protocol.

### Image Analysis

Image analysis was performed with software (MATLAB 2018b; MathWorks) using self-developed scripts and commercial toolboxes. Except for two segmentation tasks, as described in the next paragraph, all steps of analysis were performed automatically. See Appendix E1 (supplement) for details on registration and basic PREFUL analysis as per Voskrebenzev et al (5).

For image and statistical analysis, the lung parenchyma was segmented as a region of interest by manually delineating the lung boundary and excluding the large (central) vessels for each section. Additionally, a manual segmentation of a large vessel was performed. The segmentation tasks were performed by a scientist (A.V.) with more than 5 years of experience in lung MRI under supervision from a radiologist (J.V.C.) with more than 15 years of MRI experience. The averaged perfusion signal in the vessel region of interest was later used to sort images according to their cardiac phase, as described in the PREFUL perfusion analysis section. Analysis of each patient required approximately 4 hours (including registration, analysis, and segmentation of three sections).

***Quantification of RVent dynamics.—***To perform PREFUL ventilation analysis, a low-pass filter with cutoff at 0.6 Hz was applied to the registered images to remove signal variations due to perfusion. Images were sorted according to their phase by analyzing a spatially averaged lung signal with a cosine model function to create one respiratory cycle with increased temporal resolution. The sorted images were interpolated to an equidistant time grid with 60 phases, which corresponds to a nominal resolution of 55 msec considering an arbitrarily chosen respiratory rate of 0.3 Hz. The static RVent and RVent time series were calculated for each phase according to the RVent definition in Zapke et al (14).

RVent slopes, which act as an MRI surrogate for airflow, were calculated for each voxel in the lung parenchyma by applying the symmetric difference quotient (first derivative) to the RVent time series. The segmentation of the flow-volume reference region of interest was performed automatically inside the lung parenchyma region of interest based on the RVent values. Values inside the 80–90 percentile range were considered healthy. This procedure was performed for each section and each participant separately. Therefore, spatial averaging led to a reference flow-volume curve in each individual for each section. The remaining flow-volume loops covering the complete respiratory cycle in the rest of the lung parenchyma were correlated (using cross-correlation as measurement of similarity with fixed time displacement of zero) to the reference flow-volume loop to measure the similarity of the time course, creating a two-dimensional flow-volume loop correlation map (FVL-CM). A high correlation was interpreted as normal ventilation cycle and a low correlation as abnormal ventilation cycle.

***PREFUL perfusion analysis.—***Using the same registered images as in the ventilation analysis, a high-pass filter with cutoff at 0.8 Hz was applied to remove signal variations due to respiration. The cardiac phase of each image was estimated by analysis of the average signal time series in a large vessel segmentation using a piecewise sine fit, creating a retrospectively sorted cardiac cycle with increased temporal resolution. The sorted images were interpolated to an equidistant time grid with 30 phases, which corresponds to a nominal resolution of 33 msec for a heart rate of 60 beats per minute (1 Hz). The averaged signal in a large vessel segmentation of the coronal section at the middle of the tracheal level and average heart frequency of the respective acquisition were used to quantify perfusion in mL/min/100 mL according to Kjørstad et al (15).

***Ventilation-perfusion match.—***Ventilation defect percentage (VDP) and perfusion defect percentage (QDP) were calculated as outlined in Appendix E1 (supplement). VDP was calculated by applying a 90% threshold to the FVL-CM as per Moher Alsady et al (9). Areas below this threshold were considered as a ventilation defect, and by calculating the relative regions with ventilation below this threshold, the VDP could be derived. QDP was calculated by applying a 20 mL/min/100 mL threshold to Q as discussed in Appendix E1 (supplement). VQM was defined as the relative area where perfusion and ventilation threshold maps show the same value. See Appendix E1 (supplement) for further details. A voxel with ventilation and perfusion defect or a voxel with no ventilation and no perfusion defect was considered a match. Two ventilation parameters were used for VQM: RVent and FVL-CM. Thereby, in combination with Q, VQM_RVent_ and VQM_CM_ were obtained, respectively.

### Pulmonary Function Testing

FEV_1_ and forced vital capacity were measured by spirometry in accordance with American Thoracic Society/European Respiratory Society recommendations (16) and as described previously (13). Further details are available in Appendix E1 (supplement).

### Statistical Analysis

Differences in ventilation, perfusion, and VQM between IND-GLY and placebo were compared using an analysis of variance statistical model, with study treatment, patient, and period included as factors. Patient was included as a random effect. Least squares (LS) treatment means are reported as point estimates for pairwise treatment comparisons. Post hoc correlation with traditional measurements of lung function and clinical outcomes was analyzed using Pearson correlation coefficient to examine the relationship between the assessed end points. The level of statistical significance was defined as *P* less than .05. Statistical analyses were conducted by Novartis Healthcare using software (SAS, version 9.4, 2013).

### Industry Support

Novartis Pharma provided funding and IND-GLY (110/50 μg) and matching placebo for the CLAIM study and funding for this analysis. One author is an employee of Novartis Pharma. Three authors acted as consultants to Novartis Pharma on an ad hoc, as needed basis during the conduct of the study. Authors who are not employed or consultants in the pharmaceutical industry had control of the data and the information submitted for publication.

## Results

### Participants

A total of 421 patients were screened, and 62 eligible participants were randomly assigned to treatment: 30 to IND-GLY followed by placebo and 32 to placebo followed by IND-GLY. In total, 57 patients completed both treatment periods (13). Of these, 50 patients (35 men: mean age, 64.8 years [range, 46–78 years]; 15 women: mean age, 62.3 years [range, 51–73 years]) had fully complete cardiac and lung MRI data sets and are included here. In total, 359 patients were excluded from the original CLAIM study (screening failures), and 12 were excluded from this retrospective analysis, either due to early trial discontinuation (*n* = 3), protocol deviations (*n* = 2), or an incomplete MRI data set (*n* = 7) (see Fig E1 [supplement]). The incomplete protocol of the seven patients can be explained by the fact that PREFUL was an exploratory end point that required a longer measurement protocol. Therefore, seven patients either chose individually or were physically (as a result of dyspnea) unable to complete the whole protocol. Table 1 shows patient demographics and characteristics.

**Table 1:**
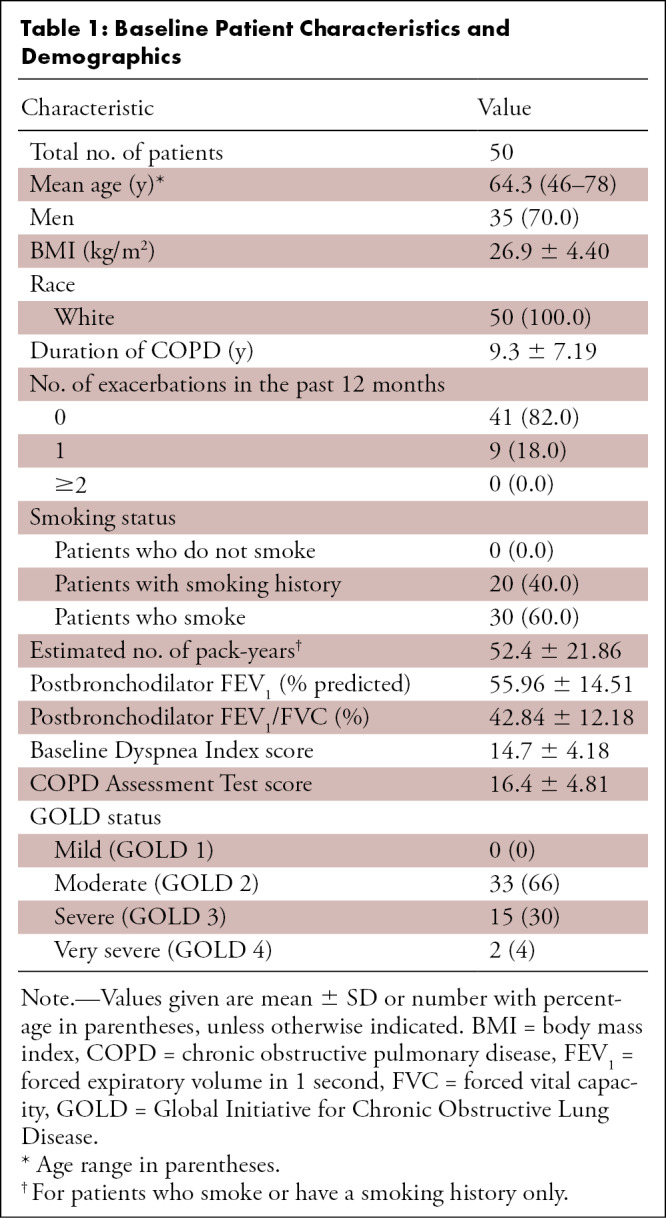
Baseline Patient Characteristics and Demographics

### Ventilation Dynamics

**Figure 1:**
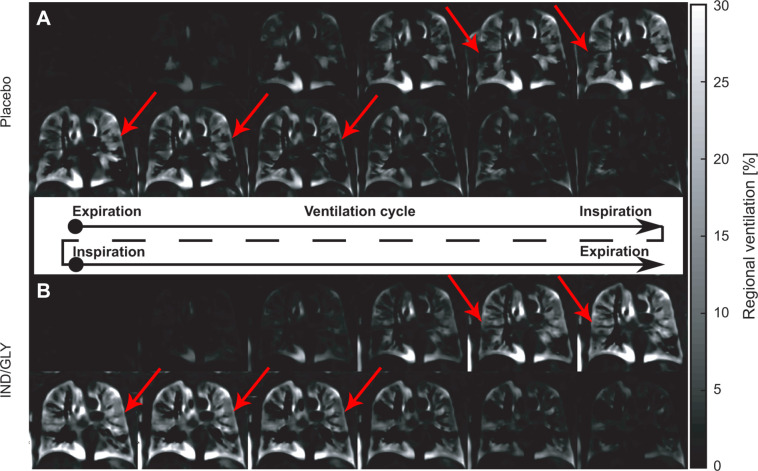
Ventilation cycle following treatment with placebo and IND-GLY in one patient. Shown is the ventilation cycle of one patient with COPD (man, 67 years old, postbronchodilator FEV_1_ at baseline of 31.6% and FEV_1_ postbronchodilator treatment of 36.7%, GOLD stage 3) following treatment with **(A)** placebo and **(B)** IND-GLY calculated with PREFUL MRI. For display purposes, the time resolution is reduced to six expiration-to-inspiration and six inspiration-to-expiration phases. Note the more homogeneous ventilation across the lungs after IND-GLY treatment as indicated by the arrows. Images were acquired without contrast agent administration using a two-dimensional gradient-echo sequence in coronal orientation. IND/GLY = indacaterol-glycopyrronium, COPD = chronic obstructive pulmonary disease, FEV_1_ = forced expiratory volume in 1 second, GOLD = Global Initiative for Chronic Obstructive Lung Disease, PREFUL = phase-resolved functional lung.

Figure 1 shows the ventilation cycle for one patient (67-year-old man, postbronchodilator FEV_1_ at baseline of 31.6%, FEV_1_ postbronchodilator treatment of 36.7%, Global Initiative for Chronic Obstructive Lung Disease stage 3) following treatment with placebo and IND-GLY obtained with PREFUL MRI. Movies 1 and 2 demonstrate this ventilation cycle.

**Figure 2:**
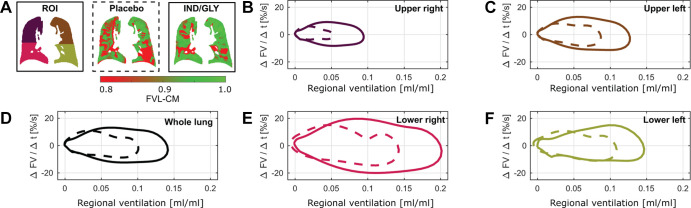
Flow-volume loops following treatment with placebo and IND-GLY in one patient. The ventilation cycles shown in Figure 1 were used to calculate regional flow-volume loops. For illustration, the averaged flow-volume loops are shown for one patient with COPD (man, 67 years old, postbronchodilator FEV_1_ at baseline of 31.6% and FEV_1_ postbronchodilator treatment of 36.7%, GOLD stage 3) for **(B, C, E, F)** four quadrants of one section and **(D)** whole-section average following placebo (dashed line) and IND-GLY treatment (solid line). **(A)** Note the lower regional ventilation values (red) and a more shallow and irregular flow after placebo versus after bronchodilator. The flow-volume loops are in concordance with reduced (red) mean flow-volume correlation map (FVL-CM) values: 0.80 versus 0.86 (whole lung), 0.83 versus 0.85 (upper right), 0.81 versus 0.78 (lower right), 0.88 versus 0.96 (upper left), and 0.58 versus 0.84 (lower left) for placebo and IND-GLY treatment, respectively. Images were acquired without contrast agent administration using a two-dimensional gradient-echo sequence in coronal orientation. Regional ventilation values ranging from expiration to inspiration (x-axis). COPD = chronic obstructive pulmonary disease, FV = flow-volume, FVL-CM = flow-volume loop correlation map, GOLD = Global Initiative for Chronic Obstructive Lung Disease, IND/GLY = indacaterol-glycopyrronium, ROI = region of interest, Δt = change in time.

**Movie 1:** Full respiration cycle after placebo. The video shows the full respiration cycle of one patient (man, 67 years old, postbronchodilator FEV_1_ 31.6%, GOLD stage 3) for one coronal section located at the tracheal bifurcation after placebo. To reduce the complexity of this dynamic information, the data are further processed to flow-volume loop correlation maps as shown in Figure 2. Please note the local artifacts, which are visible during the ventilation and perfusion cycle outside of the lung. These can be explained as follows: The registration of the image was limited to a region of interest covering the lung. Structures outside the lung can be prone to registration deformations or unregistered body movements. These residual movements, which also include out-of-plane movements, often occur at the frequency of breathing or cardiac movement. FEV_1_ = forced vital capacity in 1 second, GOLD = Global Initiative for Chronic Obstructive Lung Disease.

**Movie 2:** Full respiration cycle after treatment. The video shows the full respiration cycle of one patient (man, 67 years old, postbronchodilator FEV_1_ 31.6%, GOLD stage 3) for one coronal section located at the tracheal bifurcation postbronchodilator treatment. To reduce the complexity of this dynamic information, the data are further processed to flow-volume loop correlation maps as shown in Figure 2. Please note the local artifacts, which are visible during the ventilation and perfusion cycle outside of the lung. These can be explained as follows: The registration of the image was limited to a region of interest covering the lung. Structures outside the lung can be prone to registration deformations or unregistered body movements. These residual movements, which also include out-of-plane movements, often occur at the frequency of breathing or cardiac movement. FEV_1_ = forced vital capacity in 1 second, GOLD = Global Initiative for Chronic Obstructive Lung Disease

**Figure 3:**
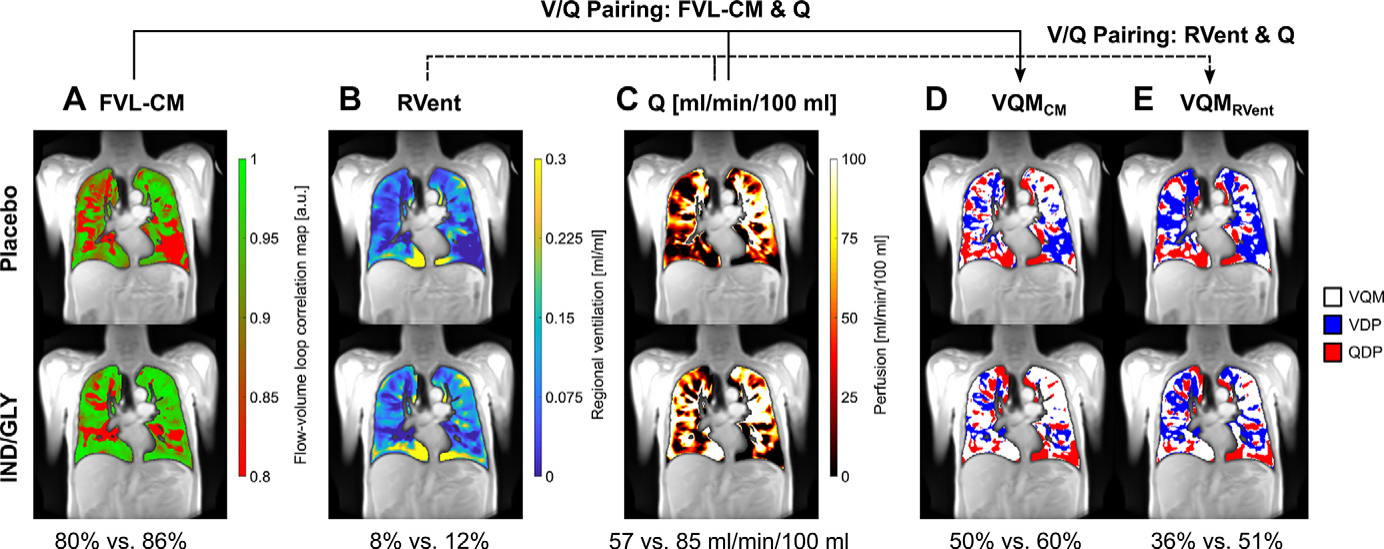
PREFUL MRI parameter maps following treatment with placebo and IND-GLY in one patient. Shown are the FVL-CM (see Figs 1, 2), RVent, Q, VQM_CM_, and VQM_RVent_ of one patient with COPD (man, 67 years old, postbronchodilator FEV_1_ at baseline of 31.6% and FEV_1_ postbronchodilator treatment of 36.7%, GOLD stage 3) for one coronal section located at the tracheal bifurcation. Note the difference between postplacebo and postbronchodilator treatment: **(A)** 80% versus 86% (FVL-CM), **(B)** 8% versus 12% (RVent), **(C)** 57 versus 85 mL/min/100 mL (Q), **(D)** 50% versus 60% (VQM_CM_), and **(E)** 36% versus 51% (VQM_RVent_), respectively. Ventilation and perfusion defects (VDP and QDP) were defined as values below threshold (Q < 20 mL/min/100 mL, FVL-CM < 0.9, RVent < 0.075). Images were acquired without contrast agent administration using a two-dimensional gradient-echo sequence in coronal orientation. COPD = chronic obstructive pulmonary disease, FVL-CM = flow-volume loop correlation map, GOLD = Global Initiative for Chronic Obstructive Lung Disease, IND/GLY = indacaterol-glycopyrronium, PREFUL = phase-resolved functional lung, Q = perfusion, QDP = perfusion defect percentage, RVent = regional ventilation, V = ventilation, VDP = ventilation defect percentage, V/Q = ventilation-perfusion, VQM = ventilation-perfusion match, VQM_CM_ = ventilation-perfusion match with Q and FVL-CM, VQM_RVent_ = ventilation-perfusion match with Q and RVent.

Figures 2 and 3 show the flow-volume loops derived from the ventilation cycle and further parameter maps calculated with PREFUL MRI for the same patient following treatment with placebo and IND-GLY. Postbronchodilator treatment values were consistently improved compared with placebo (see Fig 3 for details).

**Figure 4:**
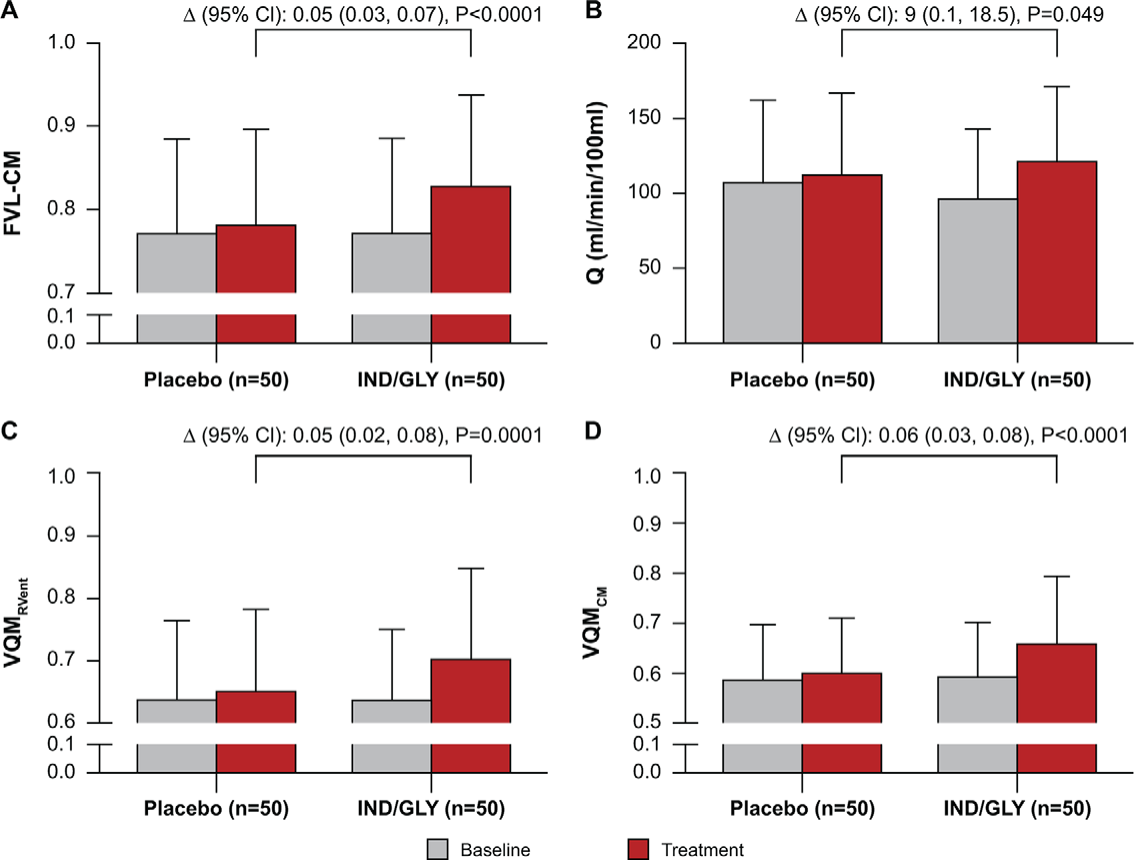
Effect of IND-GLY on FVL-CM, perfusion, and VQM (*n* = 50). Analysis of change in **(A)** FVL-CM as calculated using the mean correlation metric of the coronal sections, **(B)** Q, **(C)** VQM_RVent_, and **(D)** VQM_CM_. The ANOVA model calculated the outcome from treatment plus patient plus period with the patient included as a random effect. Δ denotes least squares means treatment differences compared with placebo. Error bars denote SD. ANOVA = analysis of variance, FVL-CM = flow-volume loop correlation map, IND/GLY = indacaterol-glycopyrronium, RVent = regional ventilation, Q = perfusion, V = ventilation, V/Q = ventilation-perfusion, VQM_CM_ = ventilation-perfusion match with Q and FVL-CM, VQM_RVent_ = ventilation-perfusion match with Q and RVent.

As shown in Table 2, in the IND-GLY treatment period, mean correlation as measured by FVL-CM changed from 0.77 ± 0.11 (SD) at baseline to 0.83 ± 0.11 after treatment, while in the placebo period this parameter changed from 0.77 ± 0.11 at baseline to 0.78 ± 0.12 after placebo treatment, representing a LS means treatment difference of 0.05 (95% CI: 0.03, 0.07; *P* < .0001) (Fig 4).

**Table 2:**
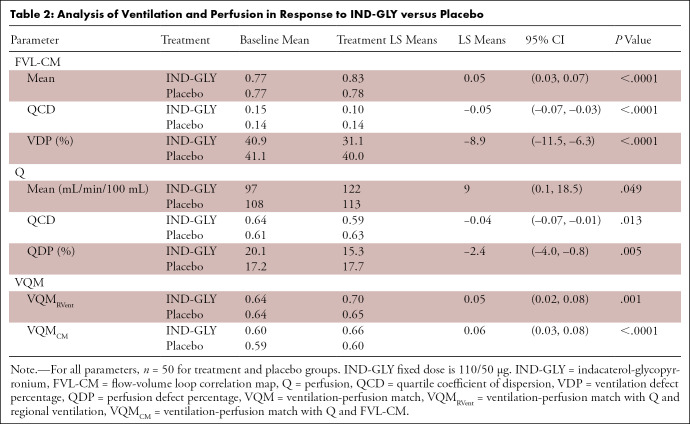
Analysis of Ventilation and Perfusion in Response to IND-GLY versus Placebo

Similar effects were observed at a regional level. Ventilation heterogeneity (as measured by quartile coefficient of dispersion) was reduced with IND-GLY treatment compared with placebo (LS means treatment difference: –0.05 [95% CI: –0.07, –0.03]; *P* < .0001) (Table 2).

IND-GLY also reduced FVL-CM–derived VDP compared with placebo (LS means treatment difference: –8.9% [95% CI: –11.5, –6.3]; *P* < .0001), which is a relative change of −28.6%. While reductions in VDP were observed with IND-GLY (40.9% at baseline to 31.1% after 14 days of treatment), results for this parameter remained largely unchanged following placebo (41.1% at baseline to 40.0% after 14 days) (Table 2). A comparatively smaller change in the measurement of hypoventilated and nonventilated regions of the lung was previously reported using static regional ventilation metrics, which included only the inspiratory and expiratory respiratory phases during free tidal volume breathing: The area of hypoventilated and nonventilated regions of the lung was decreased by 5% in response to IND-GLY (relative change of –14.3% vs placebo) (12).

### Perfusion

In the IND-GLY group, pulmonary perfusion in the parenchyma was 97 mL/min/100 mL at baseline, rising to 122 mL/min/100 mL following IND-GLY treatment. Negligible changes were observed between baseline (108 mL/min/100 mL) and placebo treatment (113 mL/min/100 mL). Patients receiving 14-day IND-GLY treatment experienced a 9 mL/min/100 mL (8.2% relative) increase in mean Q compared with placebo (LS means treatment difference: 9 mL/min/100 mL [95% CI: 0.1, 18.5]; *P* = .049) (Fig 4).

Furthermore, Table 2 shows that IND-GLY decreased quartile coefficient of dispersion of perfusion by 0.04 compared with placebo (95% CI: –0.07, –0.01; *P* = .013), indicating improved perfusion heterogeneity throughout the lung with IND-GLY treatment.

Finally, a change of 2.4% QDP was observed with IND-GLY compared with placebo (95% CI: –4.0, –0.8; *P* = .005) (Table 2).

### Ventilation-Perfusion Match

VQM_RVent_ was improved with IND-GLY treatment compared with placebo: IND-GLY increased VQM_RVent_ from 0.64 at baseline to 0.70 after treatment, whereas placebo increased VQM_RVent_ from 0.64 at baseline to 0.65 only (LS means treatment difference: 0.05 [95% CI: 0.02, 0.08]; *P* = .001) (see Table 2 and Fig 4).

More significant results were obtained for VQM_CM_. IND-GLY increased VQM_CM_ from 0.60 at baseline to 0.66 after treatment, whereas placebo only increased VQM_CM_ from 0.59 at baseline to 0.60 (Table 2) (LS means treatment difference: 0.06 [95% CI: 0.03, 0.08]; *P* < .0001) (see Table 2 and Fig 4).

### Correlation of PREFUL MRI–derived Measurements with Traditional Measurements of Lung Function and Other Cardiac and Clinical Outcomes

**Figure 5:**
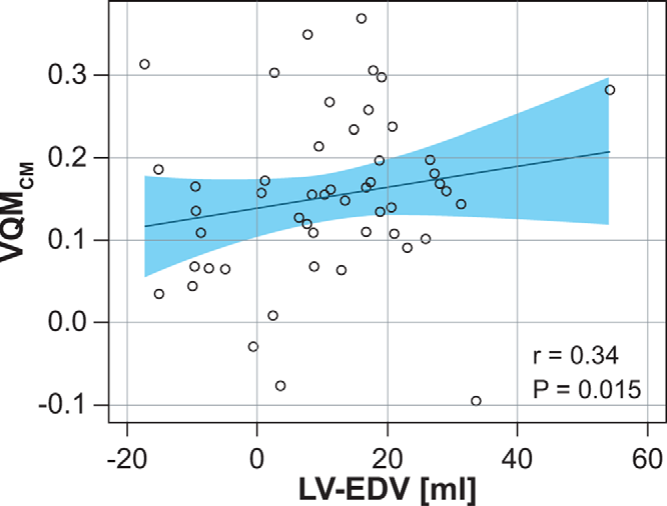
Correlation analysis of FVL-CM with left ventricular end-diastolic volume comparing changes due to treatment response. Post hoc Pearson rank correlation analyses (IND-GLY–placebo) of VQM_CM_ change with left ventricular end-diastolic volume change. Linear regression lines ± CIs are denoted. The measurement of combined positive effects on perfusion and ventilation (VQM_CM_) add up, explaining improved ventricular filling that is likely due to improved oxygenation and microvascular function. FVL-CM = flow-volume loop correlation map, IND/GLY = indacaterol-glycopyrronium, LV-EDV = left ventricular end-diastolic volume, VQM_CM_ = ventilation-perfusion match with Q and FVL-CM.

Posttreatment correlation and treatment change analyses demonstrated significant relationships between ventilation and perfusion measures with spirometry, body plethysmography, left ventricular filling, and dyspnea score measurements (Tables E1, E2 [supplement]). In particular, there were posttreatment correlations (Table E1) between FVL-CM and FEV_1_ (*r* = 0.65, *P* < .0001), which were stronger compared with RVent and FEV_1_ (*r* = 0.33, *P* = .001). Additionally, treatment changes in VQM_CM_ were correlated with left ventricular end-diastolic volume (*r* = 0.34, *P* = .015) (Fig 5; Table E2), and treatment changes in QDP were correlated with TDI (*r* = –0.31, *P* = .026; Table E2). For more details, see Appendix E2 (supplement).

## Discussion

PREFUL MRI–derived regional flow-volume loop and pulmonary perfusion measures are correlated to treatment response in patients with COPD before and after IND-GLY treatment in this retrospective analysis of the CLAIM study. The PREFUL technique identified significant treatment changes for various ventilation and perfusion parameters, as well as significant decreases in VDP and QDP, leading to increased ventilation-perfusion matching in response to IND-GLY compared with placebo. This study demonstrated that diverse patterns of flow-volume curves can be visualized in patients with COPD using PREFUL MRI, and these can be further evaluated with a correlation metric, measuring the similarity to an individualized reference flow-volume loop.

While clinical spirometry uses a forced expiratory breathing maneuver, PREFUL MRI spirometry uses resting tidal volume breathing. FVL-CM and VDP derived from FVL-CM correlates strongly with FEV_1_ changes after IND-GLY treatment. This correlation is much stronger than that between the static RVent measurement and FEV_1_. This suggests that the flow-volume loop analysis at tidal volume breathing is more closely related to FEV_1_ than the RVent measurement, which only uses the inspiratory and expiratory phase. Whether full lung coverage instead of three coronal sections or forced expiratory breathing maneuvers further improve the correlation to FEV_1_ remains to be explored.

In this study, we used the same MR image time series to derive both ventilation and perfusion parameters using different filters for perfectly aligned voxelwise comparisons, which allowed for accurate calculation of VQM; IND-GLY treatment induced significant improvements in VQM, demonstrating the treatment benefits of dual bronchodilation. MRI-based flow-volume assessment was previously demonstrated using grid-tagging MRI (17), segmentation of single-lung geometry of two-dimensional and three-dimensional acquired MRI studies (18), and registration deformation information (19). To our knowledge, this concept has never been applied previously to Fourier decomposition–related techniques on a regional level to monitor treatment changes in COPD.

In recent work, a subset of patients with COPD from the CLAIM study was used to measure the reproducibility of the PREFUL parameters by analyzing the baseline and 14-day posttreatment response in the subgroup receiving placebo first. The median coefficient of variations between the first scan and second scan for the relevant parameters showed a high reproducibility in that study: RVent of 9.7% (*P* = .25), FVL-CM of 2.3% (*P* = .09), and Q of 12.2% (*P* = .24). These findings support the differences between IND-GLY and placebo PREFUL measurements as a true treatment response. Moreover, they confirm FVL-CM as a stable parameter (11).

Post hoc correlation analyses revealed significant relationships between the PREFUL measurements described herein with parameters measured by spirometry, body plethysmography, left ventricular filling, and patient-reported outcomes. Importantly, lung function was assessed with controlled timing after medication on the same day as the MRI procedure in this study, allowing meaningful correlations to be drawn from these analyses. TDI is an important patient-reported measure of dyspnea. Here, we describe a significant posttreatment association between TDI and the correlation metric of regional ventilation (FVL-CM) and VQM_CM_, which is weaker with VQM_RVent_ and is not observed with RVent. Therefore, the correlation metric to measure ventilation may potentially reflect clinically relevant measures of dyspnea in patients with COPD. Of further interest is the significant correlation between perfusion heterogeneity and TDI. As perfusion heterogeneity improved with IND-GLY treatment (as measured using perfusion quartile coefficient of dispersion and QDP), so too did TDI, suggesting a link between perfusion heterogeneity and patient-reported dyspnea. A number of these correlations were no longer significant when treatment changes were specifically analyzed, such as in the posttreatment correlation for the correlation metric of RVent with left ventricular end-diastolic volume (the primary end point of the CLAIM study [13]). However, the combination of the suggested ventilation and perfusion parameters, as calculated by treatment change in VQM_CM_, showed a significant treatment correlation with left ventricular end-diastolic volume (*P* = .015). This seems plausible because the measurement of combined positive effects on perfusion and ventilation add up, thus explaining improved ventricular filling likely due to improved oxygenation and microvascular function.

This study carried several limitations. First, identification of a healthy flow-volume curve for reference may be challenging in patients with very severe disease, and the automatic selection relies on the assumption that high RVent corresponds to healthy lung regions. Nevertheless, this approach was shown to deliver very similar results in patients after double lung transplantation in comparison with manual segmentation of a healthy region of interest by an experienced radiologist who visually analyzed the whole respiratory cycle (9). Also, although a reference derived from a healthy population would be desirable for FVL-CM calculation, this approach is currently not practicable because the inter- and intrapatient variability (eg, thoracic vs diaphragmatic breathing) of MRI-derived flow-volume curve shapes has not yet been investigated in detail. Therefore, it is difficult to determine a correlation cutoff. Another limitation was the fact that the technique proposed herein relies on only partial acquisition to reflect the whole lung volume. Increasing spatial resolution and achieving full lung volume coverage, as demonstrated by the recently developed three-dimensional PREFUL technique, could potentially enable the measurement of even smaller treatment changes and reduce the problem of partial volume artifacts and the interrelated segmentation inaccuracies (20). Additionally, the correlation of perfusion measured by PREFUL with dynamic contrast-enhanced MRI–derived pulmonary microvascular blood flow may be imperfect because only one coronal section at the level of the trachea for dynamic contrast-enhanced MRI was compared with three coronal sections for the PREFUL analysis. A more detailed analysis was recently published (10). Furthermore, the PREFUL technique depends on a negative signal measure relative to expiratory parenchymal signal; therefore, in low signal-to-noise ratio conditions such as hyperinflated lung, the dynamic range of this measure is limited. Also, the ventilation and perfusion measurements are conducted indirectly with PREFUL (and other Fourier decomposition–related methods), relying on the MRI signal as a surrogate marker. Nevertheless, more direct measurements with fluorinated gas inhalation (4) and the reference standard SPECT (21) confirm the validity of the signal-based model. As this was a post hoc analysis, *P* values were not adjusted and therefore should be interpreted with caution. Finally, although the observed strong treatment effect of dual bronchodilation in the CLAIM study allowed investigation of the link between PREFUL MRI–based measurements with clinical outcomes, CLAIM was not designed primarily to investigate PREFUL functional MRI outcomes.

In conclusion, RVent dynamics and VQM measured by PREFUL MRI show treatment response in COPD. IND-GLY significantly improves RVent dynamics, perfusion, and VQM in patients with COPD. Additionally, the noninvasive, free-breathing PREFUL MRI method could provide important end points in future clinical cardiopulmonary trials in the form of dynamic regional assessment of lung function and information regarding the entire respiratory cycle.
